# A Science Mapping Analysis of Computational Methods and Exploration of Electrical Transport Studies in Solar Cells

**DOI:** 10.3390/ma19030452

**Published:** 2026-01-23

**Authors:** Noor ul ain Ahmed, Patrizia Lamberti, Vincenzo Tucci

**Affiliations:** Department of Information and Electrical Engineering and Applied Mathematics, University of Salerno, 84084 Fisciano, SA, Italy; plamberti@unisa.it (P.L.); vtucci@unisa.it (V.T.)

**Keywords:** science mapping, computational modeling, electrical transport, perovskite solar cells, VOSviewer, efficiency

## Abstract

**Highlights:**

**What are the main findings?**

- This study identifies the leading contributors, collaboration networks, and emerging research directions.
- The most important keywords in the field of solar cells are identified.
- Band gap engineering is necessary for optimal device performance.

**What are the implications of the main findings?**

- This study found a structured framework for identifying research trends and knowledge gaps.
- It highlights the need for careful calibration of the charge transport interface.
- It reveals modeling gaps and proposes multiphysics strategies and 3D models.

**Abstract:**

This study investigates the state of the art related to the computational methods for solar cells. Numerical modeling is a basic pillar that is used to ensure the robust design of any device. In this paper, the results of a detailed science mapping-based analysis on the publications that focus on the “numerical modelling of solar cells” are presented. The query was conducted on the Web of Science for 2014–2024, and a subsequent filtering was performed. The results of this analysis provided the answers to the five research questions posed. The paper has been divided into two parts. In the first part, the literature search began with a broad examination, and 3259 studies were included in the analysis. To present the results in a visual form, graphs created using VOS viewer software have been used to identify the pattern of co-authorship, the geographical distribution of the authors, and the keywords most frequently used. In the second part, the analysis focused on three main aspects: (i) the influence of absorber layer thickness on optical absorption and device efficiency, (ii) the role of different ETL/HTL materials in charge transport, and (iii) the effect of illumination conditions on carrier dynamics and photovoltaic performance. By integrating the results across these dimensions, the study provides a comprehensive understanding of how these parameters collectively determine the efficiency and reliability of perovskite solar cells.

## 1. Introduction

Energy is an essential need for the existence and growth of human communities. Consequently, the need for energy has increased gradually as human civilization has progressed [1]. Solar energy is a great source of renewable energy, as it is one of the most significant and abundant natural sources of energy [2]. Perovskite has emerged as a strong photovoltaic candidate due to its properties like high power conversion efficiency (PCE) and easy synthesis process [3,4]. Computational methods enable shorter times for the design, fewer efforts for prototyping, and fewer failures. One key advantage during the design process is being able to predict the behavior of a component. Developing a reliable model of the simulation can save cost and time, leading to a robust design by overcoming the conventional trial-and-error methods [5]. Numerical modeling of solar cells serves as a vital approach to optimize devices and validate physical processes through simulation instead of performing physical tests [6]. It allows us to investigate the effects of variation in material properties and cell geometry on overall device performance [7]. Different kinds of modeling techniques and software can be chosen based on the research requirements [8]. When sunlight enters the cell, it first encounters a Transparent Conducting Oxide (TCO) layer that allows light to pass through while providing a conductive path for electrons [9]. The transparent electrode on top has a powerful role in light absorption and electron collection that can affect the overall performance of solar cells [10]. Numerous studies concentrate on the role of TCEs in solar cell efficiency. Planar MAPbI_3_ solar cells were fabricated with a graphene/(PDMS) composite as a transparent electrode. Optimization of anodization time and graphene layers achieved a 15.0% PCE under AM1.5 illumination [11]. New Figures Of Merit (FOMs) for assessing Transparent Conductive Electrodes (TCEs) in solar cells were used, focusing on the balance between transmittance and sheet resistance’s impact on device performance [12]. The optimized configuration featuring three graphene monolayers on ITO-based TCO effectively minimized surface reflection and sheet resistance of 55 Ω/sq and had a high average conductance of 13.12 Ms [13]. The Solar Cell Capacitance Simulator 1-Dimensional (SCAPS-1D) model of a perovskite solar cell has shown the optimal doping concentrations of 10^21^ cm^−3^ for CdZnS ETL and 10^20^ cm^−3^ for Spiro-OMeTAD HTL. With a perovskite absorber thickness of 600 nm, the device achieved a PCE of 25.20% [14]. To the best of our knowledge, the existing literature provides an extensive examination of various materials and technologies employed in transparent electrodes for photovoltaics [15]. Nevertheless, bibliometric mapping analysis for the state of the art related numerical modeling of solar cells and its electromagnetic behavior of light is lacking in the current literature.

In this paper, an in-depth study is conducted on numerical modeling for solar cells, specifically using perovskite-based devices as the primary example. VOS viewer software (version 1.6.20 created by “Centre for Science and Technology Studies, Leiden University, Leiden, The Netherlands”) is used as an instrument for the bibliometric analysis, which reveals the research trends in the numerical modeling of solar cells. A subsequent analysis has been performed to investigate fundamental charge transport processes and the effect of incident light on perovskite solar cells. The analysis is divided into two parts. The first part of this paper will discuss, in detail, the data collection to answer the imposed research question regarding the general analysis of the study related to numerical modeling for solar cells. It answers questions related to the relevance of numerical modeling and identifies the pattern of co-authorship, the geographical and organizational distribution of the authors, and the keywords most frequently used in this field. In the second part, the sub-analysis was more focused on perovskite solar cells to stress the limited literature that appears in the search area related to the “numerical modelling of electrical transport properties of light inside solar cells”. The detailed analysis of publications based on three different research questions and the most frequently used software is presented in this part of the paper.

## 2. Materials and Methods

**Figure 1:**
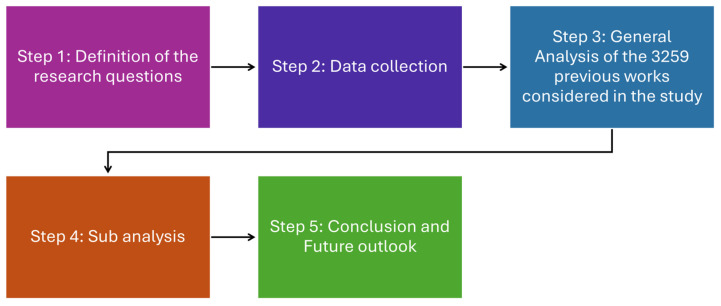
Five-step methodology.

This analysis’s approach corresponds to five steps, which are graphically presented in Figure 1 [16]. In the first step, the research questions were defined. The second step was performed to obtain data for “numerical modelling of solar cells” on the “web of science platform”. In the third step, we performed a general analysis of the studies that appeared as a result of the literature search. VOS viewer software was used as a scientific instrument to construct and visualize the bibliometric maps of authors and keywords based on co-citation and co-occurrence analysis of the data obtained. VOS viewer software refers to “Visualizations of similarities” in the obtained data. It has the possibility of displaying bibliometric maps in multiple ways and allows users to analyze the mapping analysis by evaluating the data over the years. There is a complete possibility of zooming in on the clusters and checking every detail of the author/keywords, the link strength, and the link between one item and another. The diversity of the software provided the possibility of a detailed examination of the data. To construct the maps for co-authorship patterns, the minimum number of publications, the location of the author, and their associated organization are considered. In this way, the “Figure of merit” has been established to have at least three publications by one author for the co-authorship analysis. Keyword analysis has been performed for the co-occurrence of words in the obtained Web of Science data, and the twenty most relevant keywords were extracted [17].

The sub-analysis in step 4 was more focused towards “electrical transport of light” in perovskite solar cells to realize the transport properties at the electrode–ETL/HTL interface, which is still a problem to solve in this field. Only thirty research papers appear in the search, demonstrating the limited literature in terms of electrical transport properties of perovskite solar cells. The deep analysis of the 29 papers is presented in the sub-analysis. In the 5th step, a conclusion and future perspective have been drawn based on the findings of this paper. The detailed work of each step performed will be explained in the following sections.

### 2.1. Step 1: Definition of the Research Questions

The fundamental step in any study is to define the research questions that can guide the data collection and analysis of emerging topics. To develop further work, the following five research questions were posed.

1. Is the “Numerical modelling” relevant in the field of photovoltaics?
2. What is the co-authorship pattern?
3. How are the previous works distributed geographically and organizationally?
4. What are the keyword co-occurrences in the complete dataset?
5. What are the most relevant keywords across existing studies?

### 2.2. Step 2: Data Collection

The “web of science” database core collection was used to find the answers to our imposed research questions. The first step was to check the relevance of numerical modeling in terms of solar cells.

1. Data related to “numerical modelling of solar cells” was searched in all fields without using any keyword, and a total of 3259 results appeared. This dataset corresponds to the Section 3 of this paper that is dedicated to answering the imposed research questions using VOS viewer software.
2. The “numerical modelling of electrical transport of light in solar cells” was analyzed with the keyword “perovskite”, which gives results for thirty publications.
3. One paper published before 2020 was excluded for having fewer than 5 citations, and there were twenty-nine papers left.
4. The remaining papers were kept to analyze the “electrical transport properties” of perovskite solar cells and correspond to the second part of this paper.

## 3. General Analysis of the 3259 Publications Considered in the Study

This section is structured to answer the research questions by analyzing the data obtained for “numerical modelling of solar cells”. The results were exported in txt. format with author and keyword information from the Web of Science (WOS) database.

VOS viewer software is a powerful tool used for the graphical representation of the results. Science mapping is a process used in this literature review for analysis and visualization. This technique, along with the software, is being used in a literature review in the field of photovoltaics. Mukul Kant Paliwal and colleagues map the emerging trends in the building-integrated photovoltaic system [18]. The research landscape for the perovskite solar cell was presented by Mahesh Kumar using the mapping technique [19]. This kind of analysis can be useful for the mapping of photovoltaic thermal systems [20]. The following sub-section will answer the research questions in a detailed way using VOS viewer software.

### 3.1. Is the “Numerical Modelling” Relevant in the Field of Photovoltaics?

**Figure 2:**
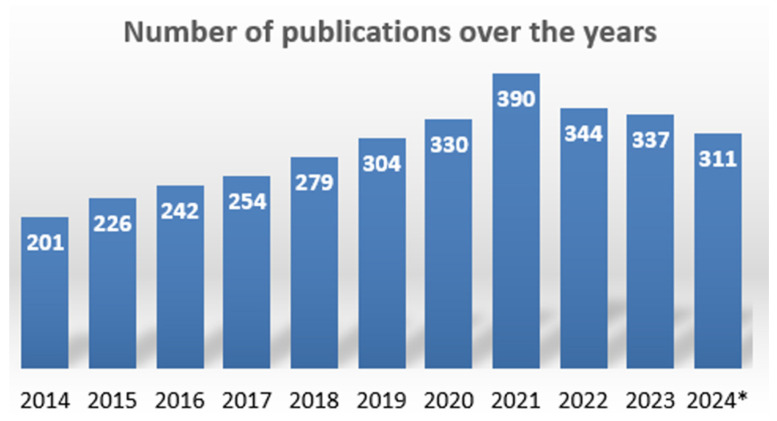
Evolution of the literature over the years (2014–2024). The * sign corresponds to the incomplete analysis for year 2024 as the study was conducted in October.

It was important to check the relevance of numerical modeling in the field of solar cells, as it can help in the design process by predicting the behavior of a device. Developing a reliable model can help the researcher overcome the conventional trial-and-error methods. By looking at step 1 of data collection, we can assume that there is a lot to discover in terms of numerical solutions in this field. Figure 2 represents the evolution of research in this field in the last ten years. The year 2024 has a * sign, as this analysis was conducted in October, which does not correspond to the complete year of 2024.

The increasing number of papers each year corresponds to the trust that researchers are developing in the modeling design. There are only a few studies reported in 2014, but the research keeps growing with each passing year, and the highest number of publications was reported in 2021.

**Figure 3:**
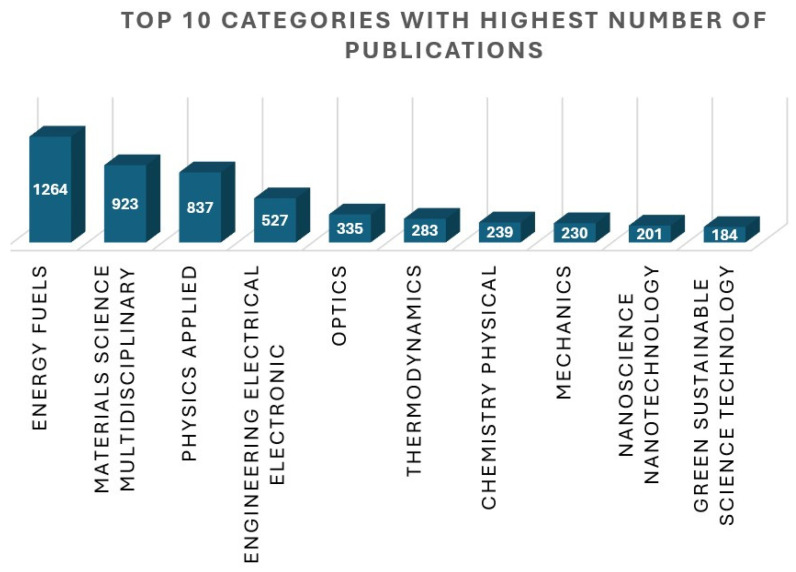
The top ten categories in the “web of science” with the highest number of publications.

Figure 3 is a visual representation of the fact that the topic is not limited to one category. It crosses many disciplines of knowledge and plays a significant part in each field of science. The top ten categories with the highest number of citations from the “web of science” are presented. The bar indicates the number of publications associated with the category mentioned on the *X*-axis. It crosses many disciplines of energy, physics, and nanotechnology. The top three categories include energy, material science, and applied physics, as depicted in the diagram.

### 3.2. What Is the Co-Authorship Pattern?

An important analysis of co-authorship patterns is needed to identify the links between different authors and their possible collaborations [21]. In VOS viewer, data is represented by clusters composed of different items. Each item represents an author. The line that connects items is called links. Two authors having joint publications are connected by links. The size of the item depends on the relevance of that specific data in the network.

Data acquired from the Web of Science was imported in VOS viewer software. A total of 11,149 authors were identified, but some authors were counted twice because of differences in name representation. Some authors used their full name in one publication and only their initials in the other one. It was important to use the thesaurus file to replace some items with others. In this way, two different papers from the same person were associated with only one author. The following two approaches were used in the thesaurus file:

1. The authors with initials were replaced by the full names of the authors.
2. The authors containing initials in both records were modified by adding a full stop at the end of their initials.

**Figure 4:**
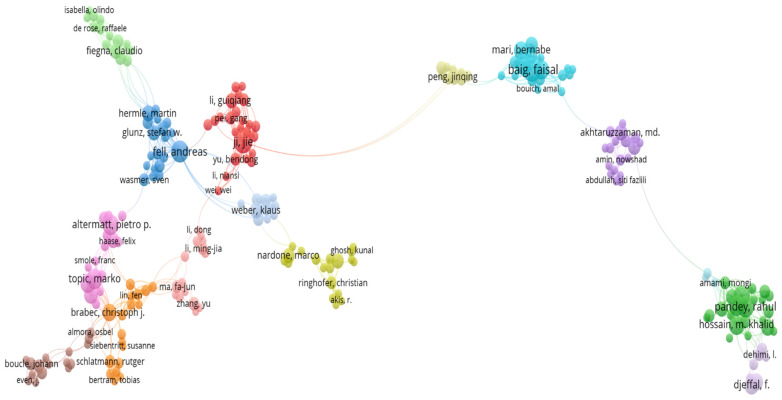
The largest set of connected authors who have at least three published studies.

After using this filtration process, the record changed to a total of 11,136 authors. Three publications were considered as a threshold for the minimum number of documents by each author. A total of 749 authors meet the threshold. Figure 4 shows the largest set of connected authors. The largest set of connected authors was 228 out of 749. The authors are shown in fifteen clusters in different colors. Each cluster represents a group of authors with the same research interest. The lines between different clusters represent the link between one group and another through collaboration or joint research work. The total number of links is 739, which presents the collaborative relations among all the authors, and the total link strength is 1695, which indicates the intensity and frequency of these connections.

**Figure 5:**
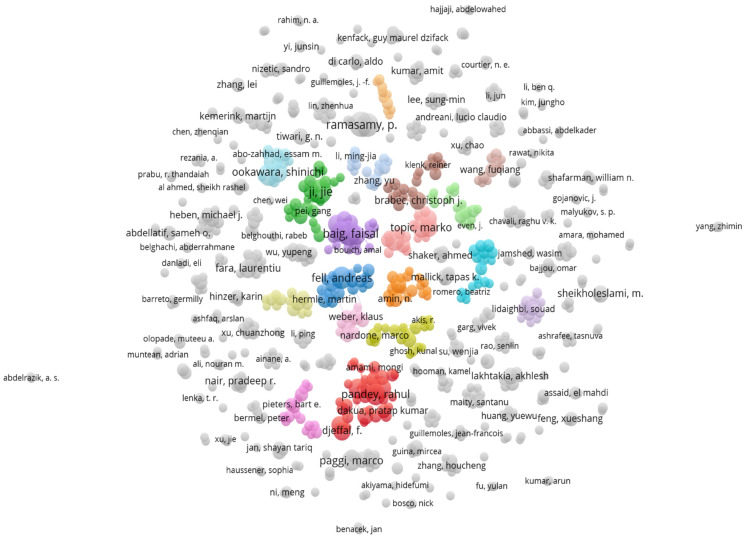
Authors with at least three studies in this field.

A map-based analysis of all the 749 authors who have at least three publications in this field is shown in Figure 5 below. The authors who do not publish joint research with others in the network are represented as isolated [22]. The total number of clusters is 194, with 1458 links and 3696 link strengths.

The deep analysis of Figure 4 and Figure 5 confirms the collaborative pattern between authors. The authors seem to work in small groups and collaborate with other research groups. Many clusters are interconnected to more than one cluster, showing interdisciplinary collaborations between researchers. Every group has one highlighted author with the greatest number of published papers, showing its major influence in numerical modeling for solar cells. The most prominent authors in this analysis are Baig Faisal and Ramasamy, P., who have the highest number of documents (twenty-one papers) published in this field. The isolated authors represented working independently and in small groups without many collaborations, who could be involved in joint research through projects, conferences, and joint publications to strengthen the research in this area.

### 3.3. How Are the Previous Works Distributed Geographically and Organizationally?

This analysis is important for understanding the collaboration pattern at the international level and the affiliation of authors. Geographical distribution analysis can find the origin of scientific activities in different parts of the world. The analysis performed for the organization can confirm the involvement of different international institutions in the study of numerical modeling of solar cells.

#### 3.3.1. Geographical Distribution

**Figure 6:**
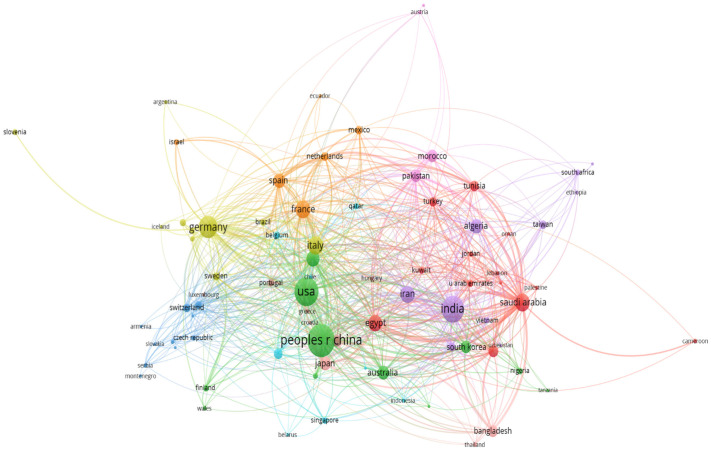
The largest set of countries that are connected to each other and published at least three studies.

Co-authorship analysis was repeated to create the geographical distribution by considering the affiliation of authors to different countries. After creating a thesaurus of data by removing the country that appears twice because of different spellings, a total of eighty-two countries were identified. Each item is a country and is linked to others if they have published something together. The following criteria were the same as the co-authorship analysis when considering those who have published at least three studies. A total of ten clusters with 743 links and 2235 link strengths were identified, and each was represented in a different color to show the countries that have published together. Figure 6 shows the largest set of countries that are connected to each other and publish at least three studies. The countries with the highest number of link strengths are the Republic of China and the United States in cluster two, represented in green color. It would be fruitful to collaborate with the countries that are not in the network to strengthen the scientific community related to the numerical methods in the field of solar cells.

#### 3.3.2. Organizational Analysis

**Figure 7:**
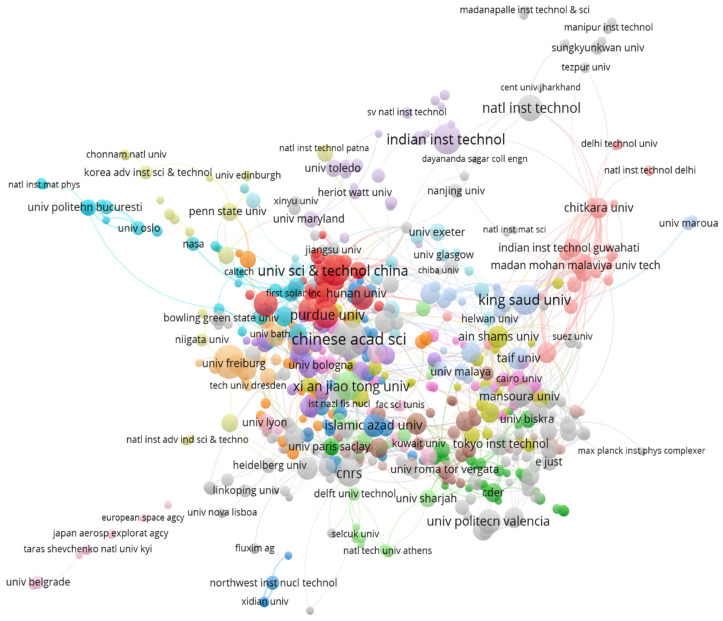
Largest set of connected organizations with at least three publications.

All studies were included to find the organizational relation for the numerical modeling of solar cells by VOS viewer software. A total of 700 organizations were identified in complete data, but after creating a thesaurus by replacing initials with full names and names of the institutions in local languages with English, the number reduced to 674. The threshold was to have at least three publications, as per previous analysis performed for co-authorship and geographic affiliations. Figure 7 shows the largest set of connected organizations having at least three studies in this field, with forty-one clusters appearing with 2014 links and 2962 link strengths. The Chinese Academy of Sciences (CAS) was found to have a maximum link strength of seventy-nine, enclosed in cluster twenty-three with fifty-eight scientific documents published. This also correlates with the fact that China is a dominant country in the geographical analysis and justifies the maximum link strength of CAS. The analysis can give insights into the groups and organizations working together on this topic. Further development of the topic can be performed by maintaining the current collaborations and involving the organizations that are currently working in isolation.

### 3.4. What Are the Keyword Co-Occurrences in the Complete Dataset?

For keyword analysis in all 3259 publications, the co-occurrence function in VOS viewer software was used by considering the full record of data from the Web of Science. A total of 3507 keywords appear, of which eighty were chosen after applying the minimum occurrence threshold of fifteen. After careful analysis of those eighty keywords, some of them seem to appear twice because of spelling and writing style differences. Two major changes were made in the thesaurus file.

1. The keyword was replaced by its plural form. For example, solar cell was replaced by solar cells.
2. Formatting styles were changed; for example, thin-film was replaced by thin film.

**Figure 8:**
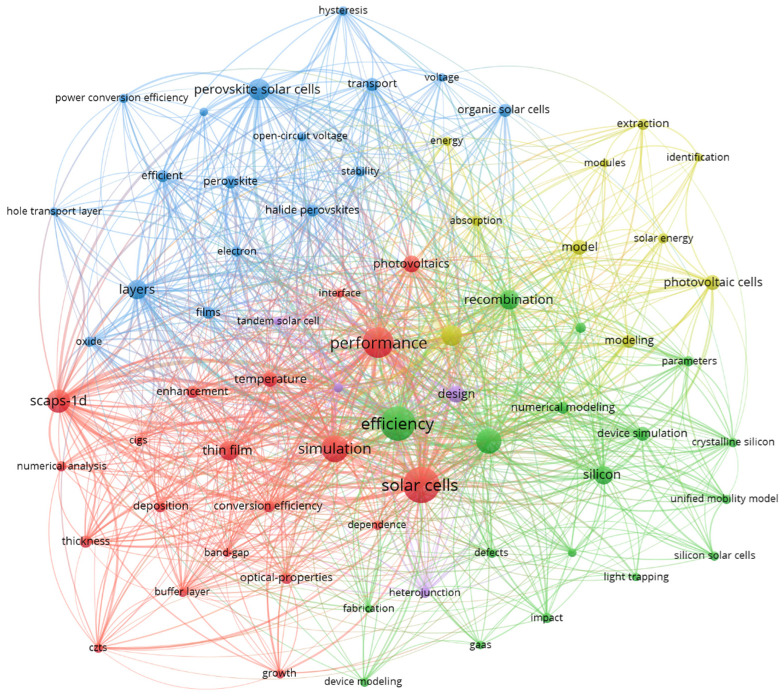
Co-occurrence of keywords in the complete dataset.

After applying the changes, the remaining sixty-nine keywords were mapped in the software, as shown in Figure 8. The keywords are grouped in five different clusters represented by distinct colors. Each cluster is led by one dominant keyword. Cluster one focuses on the keyword solar cells. Cluster two and cluster three are led by efficiency and layers, respectively. Clusters 4 and 5 are guided by optimization and design. Table 1 below shows the list of keywords that appear in each cluster and their co-occurrence. Each dominant word is in close approximation with other keywords in the clusters. They are grouped in the same cluster because of their relevance and occurrence in almost similar types of content. Table 1 below shows the list of keywords appearing in similar clusters. Cluster 1 includes 20 keywords that are explained in the table below. Clusters 2 and 3 include 18 and 17 keywords, respectively. Cluster 4 has ten keywords, and the cluster has a minimum of four keywords. Cluster 1 has the highest number of keywords, and the most prominent word is solar cells.

### 3.5. What Are the Most Relevant Keywords Across Existing Studies?

The twenty most important keywords that occurred in the dataset are presented in Table 2. Efficiency is the keyword with the highest occurrence and link strength. It is also one of the most important parameters when considering the performance of solar cells [23]. The rest of the 19 keywords are also closely related to efficiency, as there are multiple factors that can contribute to the efficiency of solar cells. An important thing to highlight here is the occurrence and link strength of the words that are closely related to the numerical modeling. Many words like simulation, numerical simulation, device simulation, design, scaps-1d are related to the numerical modeling of solar cells but appear multiple times here because of the separate ways of representation in studies. The number of times that a keyword occurs and its link strength represent its usefulness in this field. Keywords related to the simulation have a high occurrence and link strength of 153 and 358, respectively.

The systematic review presented in the previous section revealed the landscape of numerical modeling of solar cells. The number of reported studies remained low at the start, but ongoing research resulted in maximum activity in 2021. This analysis demonstrates strong interdisciplinary research spanning multiple disciplines of energy, materials science, and applied physics as the top three categories. Baig Faisal and Ramasamy P. establish themselves as the most prominent authors with 21 papers each in this domain. The Chinese Academy of Sciences (CAS) operates as the institution with the strongest research influence by maintaining 79 link strengths and publishing 58 documents. China and the United States lead as the major geographical contributors in international research networks. Research impact could receive more boost through new partnerships with institutions and countries that currently do not participate enough. The bibliometric analysis for the most relevant keywords revealed that efficiency is the most frequently occurring keyword, with a total of 244 occurrences and a link strength of 513, indicating its central role in solar cell performance. In addition to efficiency, other closely related keywords, such as power conversion efficiency, open-circuit voltage, transport, recombination, and stability, further emphasize the integral relationship between electric transport properties and overall device efficiency. The frequent co-occurrence of these terms in the analysis suggests that optimizing charge transport, which impacts carrier mobility, recombination rates, and the transport of electrons and holes, directly influences the performance and stability of perovskite solar cells. These findings highlight the essential role of electric transport properties in driving the efficiency of perovskite-based devices, which will be explored in detail in the upcoming section.

## 4. Sub-Analysis for the Investigation of Electric Transport Properties of Solar Cells

This section corresponds to the second part of the paper, to analyze the state of the art related to the computational methods to study the electromagnetic behavior of light inside solar cells. The existing literature provides an extensive examination of various materials and technologies employed in transparent electrodes for photovoltaics [24,25]. Nevertheless, there is still a need for a comprehensive understanding of the electrical transport properties of two-dimensional (2D) nanomaterial-based thin films and their robust design for photovoltaic applications, as it is lacking in the current literature. To bridge this research gap, we started by analyzing the scientific literature related to the development of computational methods to study the electromagnetic behavior of light inside solar cells. We initiated with the “web of science” database for comprehensive coverage.

### 4.1. Definition of Research Questions

To advance the understanding of electromagnetic (EM) behavior of light within solar cells, it is essential to examine how structural and material parameters influence optical absorption, carrier transport, and overall device efficiency [26]. Guided by this objective, the present sub-section formulates three key research questions to direct the analysis of computational studies on perovskite solar cells.

1. RQ1: How does absorber layer thickness affect light absorption and device efficiency in solar cells?
2. RQ2: What impact do different ETL/HTL materials have on performance?
3. RQ3: How do light intensity and illumination conditions influence carrier dynamics and photovoltaic efficiency?

### 4.2. Study Steps

1. We started with the “web of science” database for comprehensive coverage and searched for published results for “numerical modelling of electrical transport of light in solar cells” (all fields) in October 2024 with a time window of 2014–2024 using the keyword perovskite in all fields.
2. Thirty publications appear in total because of the search.
3. We omitted the papers with fewer than five citations published before 2020 because they might have less impact. One paper was omitted, and twenty-nine papers were left that are deeply analyzed in Section 4.3.
4. By looking at the title, abstract, and keywords of twenty-nine papers, all twenty-nine papers were selected that refer to the modeling of perovskite solar cells.
5. One article is currently in the “Article in Press” stage and is not the final “Version of record” and is not included in the analysis [27].
6. The remaining 28 papers are deeply analyzed in terms of the effect of light on the electric transport properties of perovskite solar cells.
7. In this section, we have stressed the limited literature that appears in the search area related to the electrical behavior at electrodes or the interface.

### 4.3. Deep Analysis of 28 Papers Considered in the Study

A comprehensive analysis of 28 selected research papers was carried out to address the three formulated research questions (RQ1–RQ3). The following sections present detailed answers to each question, highlighting key findings and trends observed across the reviewed studies.

Upon careful analysis of the selected 28 studies, we realize that two papers [28,29] do not report the use of perovskite material. Both papers appeared in our analysis because of the keyword “perovskite” we have used (as explained in the methodology section). The keyword search does not search for material used in the study; rather, it confirms the presence of the word in the manuscript somehow. For clarity and completeness, we have also included those papers in our discussion to address the RQs. Table 3, Table 4 and Table 5 are divided into two sections to create a boundary between perovskite and non-perovskite materials. The discussion of non-perovskite materials with respect to RQs is also presented in the corresponding sections of the tables. This analysis also highlights the limitations of the keyword search for bibliometric analysis.

#### 4.3.1. Results for RQ1: Effect of Absorber Thickness on Light Absorption and Photovoltaic Efficiency

A consistent theme across the reviewed studies is that absorber thickness directly governs the trade-off between optical absorption and charge collection. All simulations and experimental analyses show that device efficiency improves as the absorbing layer becomes thick enough to absorb longer-wavelength photons but falls once carrier diffusion limits are exceeded and recombination dominates.

Single-junction devices exhibit optimum thicknesses in the range of 100–600 nm. For example, Ahmed et al. [14] and Kumar et al. [30] both reported peak efficiencies at ~600 nm, with PCE values around 24–25%. Similar trends were seen for lead-free materials. Muniandy et al. [31] and Hamzah et al. [32] found maximum PCE at ~400 nm for MASnI_3_ and FAMASnGeI_3_, respectively. Shukla et al. [33] also confirmed that ~400 nm is optimal for FASnI_3_, provided carrier lifetimes are sufficiently long.

In contrast, Sharma et al. [34] observed a monotonic increase up to 550 nm in MASnI_3_, where efficiency remained limited by intrinsic recombination losses. In addition to these, Dzifack Kenfack et al. [35] reported that MASnI_3_ devices benefit from thicker absorbers around 1 µm, achieving ~25.31% PCE. Similarly, Samaki et al. [36] demonstrated optimum performance at ~400 nm for RbGeI_3_, with passivation strategies further boosting efficiency to nearly ~29.71%.

Indoor and wide-bandgap devices exhibited slightly different behavior. Manjhi et al. [37] demonstrated that Cs_3_Sb_2_Cl_x_I_9−x_ absorbers require thicker layers ~0.8 µm to achieve strong visible absorption under indoor spectra, with efficiencies reaching >40% under WLED light. Tandem and bilayer architectures require asymmetric absorber thicknesses to ensure current matching. Singh et al. [38] reported optimal tandem operation with 195 nm for the top cell and ~1.2 µm for the bottom cell, yielding ~31.55% efficiency. Soldera et al. [39] similarly found that bottom cell thickness in all-perovskite tandems should be 800 nm and 401 nm for the bottom and top cell, respectively, depending on optical losses in the contacts. Bhattarai et al. [24] and Baruah et al. [40] showed that graded and dual-absorber stacks achieve maximum performance when each sub-layer is ~0.6–1.0 µm thick, with PCE values exceeding 33–34%. Prashanthi et al. [41] also reported ~1 µm as optimal for dual Cs_2_BiAgI_6_/CIGS absorbers, with ~36.36% efficiency.

Finally, light management strategies can shift these thickness requirements. Hossain et al. [42] demonstrated that nanohole and nanodome front contacts allowed near-unity quantum efficiency at ~450 nm, boosting PCE to ~23.3% in single-junction PSCs and ~31% in tandem devices with thinner absorbers. Similarly, for non-perovskite materials, Cao et al. [29] optimized Sb_2_S_3_ indoor cells under fluorescent and LED lighting, but they showed that in defect-free cases, absorption continued improving up to ~2 µm, with efficiencies exceeding 43% (ideal case). These results also highlight the dependence of optimal thickness on illumination conditions. Manjhi et al. [28] demonstrate that increasing the absorber thickness initially improves performance up to 0.5 µm. Beyond this thickness, bulk recombination takes over. The optimal device performance is reached at an absorber thickness of 1.4 µm (under low defect density circumstances), resulting in maximum efficiency.

Among tandem solar cells, many studies demonstrate contrasting performance factors, although they use the same absorber family as a top cell in tandem configuration. In [41], Cs_2_AgBiI_6_ is paired with CIGS as the bottom absorber in a tandem-inspired architecture. CIGS has a very high absorption coefficient, superior carrier mobility, and lower recombination losses due to optimized defect density, which enables efficient near-infrared photon harvesting and balanced charge generation and collection. Additionally, the band alignment between Cs_2_AgBiI_6_ and CIGS facilitates effective charge separation with reduced interfacial recombination, leading to a higher simulated PCE (~36.36%) under optimized thickness conditions. In [40], the FA_0.75_Cs_0.25_SnI_3_ absorber is used as the bottom cell. This is a narrow-bandgap absorber, and its performance is intrinsically limited by higher defect densities, Sn_2+_ oxidation-related recombination losses, and lower carrier stability compared to CIGS. As a result, despite careful SCAPS-based optimization of transport layers and thickness, the achievable PCE is slightly lower (~34.01%). The MASnI_3_ absorber is used in many studies, but it gives different PCE based on its thickness and combination with other charge transport layers. The relatively low PCE of 5.42% [34] can be attributed to the high susceptibility of MASnI_3_ to Sn_2+_ oxidation, which leads to defect formation and enhanced recombination losses. Additionally, interface properties associated with PEDOT:PSS as the HTL limit further performance improvement. In contrast, Dzifack Kenfack et al. [35] report a substantial efficiency enhancement using a thicker 1 um MASnI_3_ absorber and achieving a PCE of 25.31%, which further increases to ~28% under defect-free absorber assumptions. The thicker absorber allows better light harvesting and thus more charge carrier generation and extraction. Instead of conventional TiO_2_ and Spiro-OMeTAD/PEDOT:PSS transport layers, the study employs single-walled carbon nanotubes as the electron transport layer and Cu_2_O as the hole transport layer, which enables more efficient hole transport owing to its favorable valence band alignment with MASnI_3_, which mitigates hole accumulation at the absorber/HTL interface and suppresses interfacial recombination losses. This improvement is reflected in a marked increase in the open-circuit voltage, reaching approximately 1.00 V, compared to PEDOT:PSS-based architecture. Simultaneously, CNTs are used as the ETL provides high electron mobility and reduced parasitic absorption, resulting in enhanced photocurrent densities exceeding 34 mA cm^−2^. Moreover, the optimized band alignment across the CNT/MASnI_3_/Cu_2_O interfaces promotes balanced charge carrier transport throughout the device and prevents excessive electron accumulation, thereby suppressing current saturation effects. Collectively, these transport layer-driven improvements account for the significantly higher simulated power conversion efficiencies. The same absorber is used by [31], and performance reached up to 27.97 PCE%. The authors pair 400 nm thickness with NiO HTL, which allows favorable band alignment and thus higher PCE.

These studies reveal the following:

- Optimal absorber thickness in single-junction perovskites is typically 400–600 nm.
- Indoor and wide-bandgap absorbers benefit from thicker layers of ~0.8 µm.
- Tandem and bilayer designs require asymmetric or multiple absorbers with thicknesses of ~0.2–1.2 µm for current matching.
- Optical/light-trapping approaches reduce the required thickness by enhancing absorption in thinner films.

It is worth noting that the optimal absorber thickness strongly depends on overall device configuration, interface properties, and chosen defect density. To provide clarity, Table 3 lists the absorber thickness ranges, optimum values, and theoretical efficiencies predicted by numerical simulations reported by each study, together with the reference numbers assigned in this review.

**Table 3:** Summary of reported studies on solar cells based on perovskite and non-perovskite materials addressing RQ1.

Ref	Device Structure	Thickness Range	Optimum Thickness	Maximum PCE Achieved (Theoretically)
[37]	FTO/TiO_2_/Cs_3_Sb_2_Cl_x_I_9−x_/poly-TPD/Au	0.05–2 µm	0.8 µm	PCE ~19% (45% optimized)
[41]	ITO/TiO_2_/Cs_2_BiAgI_6_ + CIGS/NiO/Au	0.2–1.6 µm	1 µm	PCE ~36.36%
[40]	ITO/TiO_2_/Cs_2_AgBiI6/Fa_0.75_Cs_0.25_SnI_3_/CuSCN/Ag	0.2–1.4 µm	0.8 + 0.6 µm bilayer	PCE ~34.01%
[39]	All-perovskite tandem (2T) Cs_0.05_FA_0.8_MA_0.15_PbI_2.55_Br_0.45_ (top cell) (FASnI_3_)_0.6_(MAPbI_3_)_0.4_	300–2000 nm (bottom cell) No range reported (top cell)	800 nm (bottom cell) 401 nm (top cell)	PCE ~32–34%
[24]	ITO/C_60_/MAPb (I, Cl)_3_ or MASnI_3_/Spiro-OMETAD/Au (single and graded double absorber)	0.2–1 µm	1 µm	Double: ~33.53% Single (MAPb (I, Cl)): 26.75%
[38]	Tandem: FA-Cs-Pb(I,Br)_3_ (top) + FA-MA-Pb-Sn-I_3_ (bottom)	Top: 0.1–1 µm; bottom: 0.1–1 µm	195 nm (top) + 1.2 µm (bottom) tandem	PCE ~31.55% tandem
[42]	FTO/TiO_2_/MAPbI_3_/Spiro-OMETAD/Au tandem (MAPbI_3_ + MASnPbI_3_), nanophotonic contacts	150–450 nm 150–300 nm (top cell) 400–1000 nm (bottom cell)	Single: 450 nm; tandem: 200–220 nm top + 800 nm bottom	31% (tandem), 23.3% (single)
[36]	FTO/TiO_2_/RbGeI_3_/HTL/Ag	200–600 nm	200–400 nm	PCE ~18% (29.71% with passivation)
[35]	TCO/CNT/MASnI_3_/Cu_2_O	0.1–1 µm	1 µm	PCE ~25.31%
[30]	FTO/TiO_2_/IL1/Cs_2_BiCuI_6_/IL2/Spiro-OMeTAD/Au	100–1000 nm	600 nm	PCE ~24.8%
[32]	FTO/CdS/FAMASnGeI_3_/NiO/Ag	100–500 nm	400 nm	PCE ~22.69%
[14]	ITO/CdZnS/CH_3_NH_3_Pb(I,Br)_3_/Spiro-OMeTAD/Au	100–1000 nm	600 nm	PCE ~25.20%
[31]	FTO/NiO/MAPbI_3_ or MASnI_3_/ZnO/Ag	50–500 nm	400 nm	PCE ~24.94% (MAPbI_3_), 27.97% (MASnI_3_)
[33]	Glass/ITO/PEDOT:PSS/FASnI_3_/C_60_/Ag	~125–1000 nm	400 nm	PCE ~17.33%
[34]	ITO/PCBM/MASnI_3_/PEDOT:PSS	50–550 nm	550 nm	PCE ~5.42%
Non-perovskite-based absorber material				
[28]	ITO/NiOx/BiOI/ZnO/Cr/Ag	0.1–2.0 µm	~1.4 µm	~12.3% (low defect), up to ~40% under WLED
[29]	FTO/SnO_2_/ZnOS/Sb_2_S_3_/Spiro-OMeTAD/Au	0.1–2.4 µm	0.25–0.35 µm indoor; ~1.5–2.0 µm ideal	PCE 22–25% (indoor) and 43–46% (ideal)

#### 4.3.2. Results for RQ2: Impact of Different ETL/HTL Materials on Performance

The choice of the electron transport layer (ETL) and the hole transport layer (HTL) has a great influence on charge extraction, recombination, and overall photovoltaic performance.

For HTLs, a consistent replacement of conventional HTL materials with inorganic alternatives, such as Cu_2_O [34,35,37,43], NiO [31,32,38,41], CuI [36], CZTS [44], Spiro + p-Si NPs [45], and CuSCN [40], results in higher PCEs and improved stability. NiO [31,32,38,41] and Cu_2_O [34,35,37,43] appear particularly versatile, delivering strong performance across both lead-free and lead-based absorbers, while CuI [36] and CuSCN [40] provide superior hole mobility and better alignment in Sn- and Bi-based perovskites. In several cases, inorganic HTLs nearly doubled the efficiency compared to devices with Spiro-OMeTAD and PEDOT: PSS [34,44]. Similarly, for non-perovskite-based absorbers, SnO_2_/ZnOS-based ETL material is proven to perform better than CdS, SnO_2_ [29].

The most conventional ETL, TiO_2_, is widely used among many studies [40,41] (as the most optimum ETL) and as a baseline [36,44,45]. In contrast, other studies outperform TiO_2_ using non-conventional materials, such as ZnO_0.25_S_0.75_ [37], MZO [38], CdS [32,43], CNT [35], CdZnS [14], and PCBM [34]. The most important thing is to find the best band alignment according to the absorber and the corresponding HTL. One optimum ETL might not stand out as best if not paired with optimal band alignment and interface properties.

Taken together, these studies demonstrate that transport layer engineering, both through material substitution and thickness optimization, is one of the most effective strategies for enhancing charge transport and suppressing recombination. Table 4 summarizes the reported ETL/HTL comparisons and the corresponding device efficiencies. Only one study [45] corresponds to experimental PCE, while the rest of the column reports theoretical/numerically simulated PCE.

**Table 4:** Summary of reported studies on perovskite on solar cells based on perovskite and non-perovskite materials addressing RQ2.

Ref	ETL	HTL	Theoretical PCE
[37]	ZnO_0.25_S_0.75_ > TiO_2_	Cu_2_O > poly-TPD	45.1% (ideal indoor), 38.7% (practical indoor)
[41]	TiO_2_ > ZnO, SnO_2_, WS_2_, C_60_, Gr-TiO_2_	NiO > Spiro, PTAA, PEDOT:PSS, Cu_2_O, etc.	36.36%
[40]	TiO_2_ > C_60_, PCBM, CeO_2_, IGZO, WS_2_, ZnO	CuSCN > Spiro, PEDOT:PSS, CuI, Cu_2_O, etc.	33.45%
[38]	MZO > C_60_, TiO_2_, SnS_2_, CdS, IGZO, PCBM, SnO_2_, STO, WS_2_, ZnO	NiO (top-cell), Zn_2_P_3_ (bottom cell) > CBTS, Cu_2_O, CuI, CuO, V_2_O_5_, CuSbS_2_, P3HT, PEDOT:PSS, Sb_2_S_3_, Spiro-OMeTAD, SrCu_2_O_2_	31.55%
[32]	CdS > TiO_2_, SnO_2_	NiO > Spiro/PEDOT:PSS	30.05%
[31]	ZnO (baseline)	NiO > Spiro	27.97% (MASnI_3_), 24.94% (MAPbI_3_)
[35]	CNT > TiO_2_	Cu_2_O > Spiro	25.31% (28% defect-free)
[14]	CdZnS > TiO_2_, ZnO, SnO_2_, etc.	Spiro-OMeTAD (optimized doping)	25.20%
[43]	CdS > TiO_2_	Cu_2_O > Spiro/PEDOT:PSS	25.02% (26.28% low defects)
[44]	TiO_2_ (baseline)	CZTS > Spiro, PEDOT:PSS, PTAA, P3HT	22.7%
[36]	TiO_2_	CuI > Cu_2_O > CuSCN > Spiro-OMeTAD	18.10% (CuI)
[45]	TiO_2_ (baseline)	Spiro + p-Si NPs > Spiro	18.7% (experimental)
[34]	PCBM > TiO_2_, MZO, C_60_	Cu_2_O > PEDOT:PSS	13.71% (11.5% baseline)
Non-perovskite-based absorber material			
[29]	SnO_2_/ZnOS > CdS, SnO_2_	Spiro-OMeTAD	26.52% (ideal), 21.0% (LED), 11.9% (AM1.5G)

#### 4.3.3. Results for RQ3: Influence of Light Intensity and Illumination Conditions on Carrier Dynamics and Photovoltaic Efficiency

The optical response and charge transport behavior of perovskite solar cells (PSCs) are strongly influenced by illumination conditions, which directly affect carrier generation, recombination kinetics, and overall power conversion efficiency (PCE) [46]. Across the reviewed literature, studies have employed both light intensity sweeps and illumination geometry variations to understand how PSCs respond under real and simulated environments.

Light intensity variation consistently showed a direct correlation with short-circuit current density (J_sc_) and a logarithmic increase in open-circuit voltage (V_oc_). Muniandy et al. [31] observed that increasing the illumination from 1 to 10 kW m^−2^ enhanced both J_sc_ and V_oc_, though the fill factor (FF) declined slightly at the highest flux due to series-resistance losses. Similarly, Tooghi et al. [47] and Manjhi et al. [37] confirmed that higher irradiance (200 → 1000 W m^−2^ and 0.2 → 1 sun, respectively) improved carrier generation, with V_oc_ following the expected logarithmic trend $V_{oc}\propto ln(I_{light})$. At lower intensities, recombination dominated, reducing charge collection efficiency.

Hamzah et al. [32] show that the photocurrent dropped by about 75% as illumination decreased from 1000 to 251 W m^−2^, yet the voltage and fill factor (FF) remained nearly constant, indicating recombination-limited behavior at weaker light. Likewise, Jacobs et al. [48] linked the buildup of large photo-capacitance under bright light to ion migration processes, showing how illumination not only governs electronic but also ionic dynamics within hybrid perovskites.

Spectral and wavelength-dependent illumination further clarified depth-specific carrier processes. Wang et al. [49] demonstrated that blue light (470 nm) generated carriers mainly near the TiO_2_/perovskite interface, resulting in slower recombination (τ ≈ 0.7–1 ms), whereas red light (620 nm) induced faster bulk recombination (τ ≈ 0.4–0.5 ms). This highlights how photon energy distribution influences charge carrier lifetime and recombination pathways.

Illumination geometry and optical transmittance also play an important role. Kenfack et al. [35] and Samaki et al. [36] showed that increasing front-contact transmittance and back-contact reflectivity substantially improved photon absorption and J_sc_, confirming that optical management directly enhances carrier generation. Furasova et al. [45] introduced p-doped Si nanoparticles within the hole transport layer, improving light trapping and boosting both J_sc_ and V_oc_ under AM1.5G conditions.

Finally, continuous monochromatic illumination experiments by Regaldo et al. [50] under a 532 nm laser exposed slow surface photovoltage relaxation (~700 s), indicating photo-induced charge redistribution and ion migration effects that persist after light removal, an important factor in PSC stability.

For non-perovskite materials, indoor and low-light illumination studies reveal re that PCE decreases with lower irradiance but can recover under spectra better matched to the perovskite bandgap, such as white LED or fluorescent light, where the spectral overlap enhances charge generation even at a few watts per square meter.

Overall, these studies confirm that both light intensity and illumination conditions critically shape perovskite solar cell operation. Increasing illumination typically boosts photocurrent and efficiency until limited by recombination or series resistance, while illumination spectrum and geometry determine where and how carriers are generated and lost. Understanding these dependencies is key to optimizing device performance under diverse outdoor and indoor lighting environments. In Table 5, the effect of variations in light intensity and light trapping configuration is depicted on J_sc_, considering its linear dependency.

In summary, the following conclusions can be made:

- A total of 100% front contact transmittance can be optimal for higher J_sc_ (33–34 mAcm^−2^), both in theoretical [35] and experimental [36] points of view.
- J_sc_ is linearly proportional to light intensity, as 10 suns can result in up to 10 times higher current density.
- Despite identical illumination, differences in device structure can lead to variations in J_sc_ due to changes in effective light trapping, parasitic losses, and charge extraction.

**Table 5:** Summary of reported studies on solar cells based on perovskite and non-perovskite materials addressing RQ3.

Ref.	Sweep Parameter	Sweep Range	Effect Observed	Optimum Illumination Condition	Performance at Optimum
[35]	Front-contact light transmittance	20% → 100%	Photocurrent and efficiency increased with higher transmittance	100% transmittance (maximum illumination)	J_sc_ = 34.65 mA cm^−2^
[36]	Front-contact transmittance and back-reflection	Transmission 20–100%; reflection 20–100%	Higher transmittance and reflection improved photon absorption and carrier generation	100% front transmission and 100% back reflection	J_sc_ = 33.51 mA cm^−2^; (experimental)
[31]	Light intensity	1 → 10 kW m^−2^	J_sc_ and V_oc_ increased with light intensity; FF slightly decreased beyond 1 kW m^−2^	10 kW m^−2^ (highest illumination)	MASnI_3_: J_sc_ = 32.05 mA cm^−2^; MAPbI_3_: J_sc_ = 27.93 mA cm^−2^;
[32]	Light intensity	1000→ 251.19 W m^−2^	J_sc_ decreased ≈ 75%; PCE dropped 6.72%; minor change in V_oc_ and FF	1000 W m^−2^ (AM1.5 illumination)	J_sc_ = 27.77 mA cm^−2^;
[47]	Light intensity	200 → 1000 W m^−2^ (AM1.5G)	Higher light intensity improved carrier generation and PCE; V_oc_ increased logarithmically with intensity	1000 W m^−2^ (1 sun)	J_sc_ = 22.88 mA cm^−2^
[45]	Incorporation of p-doped Si nanoparticles (light trapping)	AM1.5G (100 mW cm^−2^)	Enhanced light absorption and hole transport; improved J_sc_ and V_oc_	AM1.5G illumination with p-Si NPs embedded in HTL	J_sc_ = 22.02 mA cm^−2^
[37]	Light intensity	0.2 → 1 sun (AM1.5G)	Increased light intensity enhanced carrier generation, J_sc_, and PCE; V_oc_ increased logarithmically	1 sun illumination (AM1.5G, 1000 W/m^2^)	J_sc_ = 21.7 mA cm^−2^
[50]	Illumination (dark → continuous 532 nm laser)	Dark → ≈ 8 W cm^−2^	Continuous light exposure caused significant Surface Photovoltage (SPV) buildup and slow decay from photo-induced charge redistribution and ion migration	Continuous 532 nm illumination (~8 W cm^−2^)	SPV = +500 mV (TiO_x_), −320 mV (NiO_x_); decay ≈ 700 s
[48]	Illumination (dark → 1 sun)	Dark → 1 sun	Photo-illumination induced large capacitance and negative capacitance from ion migration	1 sun illumination	Photo-capacitance ≈ 10^−1^ F cm^−2^ (low frequency)
[49]	Light intensity and illumination wavelength	<1 sun → simulated >10 suns; 470 nm (blue) → 620 nm (red)	τ_TPV increased at lower light intensity (RC-limited <1 sun); red light caused faster bulk recombination; blue light slower interface recombination	>10 suns (simulated illumination); blue light (470 nm)	τ_TPV ≈ 2τ_SRH (~30 ns simulated); τ_TPV = 0.7–1 ms (blue), 0.4–0.5 ms (red)
Non-perovskite-based absorber material					
[28]	Light intensity and illumination type	200 → 1000 lux; WLED, CFL, halogen	Higher light intensity increased carrier generation; CFL/WLED gave best performance with BiOI due to higher visible-range power density, matching with BiOI absorption	1000 lux WLED illumination	J_sc_ = 1.83 mA cm^−2^
[29]	Illumination condition (AM1.5 → indoor light)	AM1.5 (1000 W m^−2^) → indoor LED/FL (1000 lux ≈ 3 W m^−2^)	J_sc_ and V_oc_ decreased under low light; FF improved; PCE was higher under LED due to spectral matching	Indoor cold-white, fluorescent light (6500 K, 1000 lux)	J_sc_ = 0.116 mA cm^−2^;

### 4.4. Software-Based Analysis of the 28 Papers Considered in the Study

**Figure 9:**
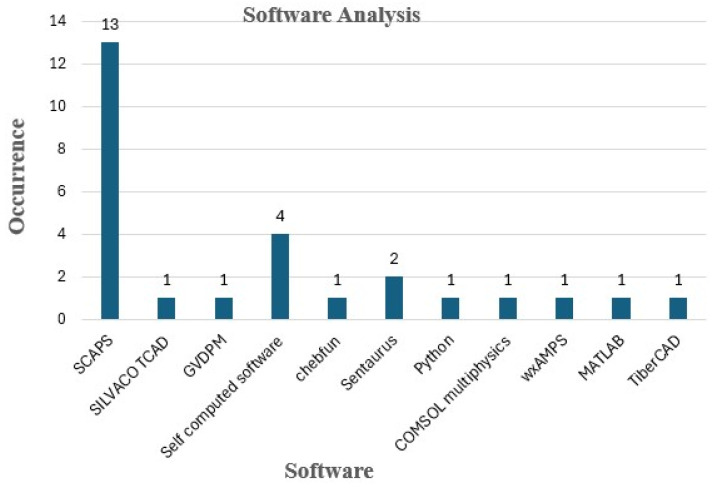
Software analysis for the obtained data from 28 studies.

A software analysis has been performed to understand the different software used for the numerical modeling of solar cells. Complete datasets, including all 28 papers, have been used to obtain this analysis to figure out the most used software for the computation of solar cells. Figure 9 shows eight different software and self-computed techniques that have been widely adopted among researchers for the analysis. The most used software is SCAPS-1D, with the highest occurrence of being used in 13 studies [14,24,28,32,34,35,36,37,38,40,41,43,44]. The self-computation technique is being adapted in four studies [42,47,49,50]. COMSOL Multiphysics software Version 6.4 can be a good candidate to be used for self-computed code. The rest of the software is used twice [33] or once in each publication.

The dominance of SCAPS-1D can be primarily attributed to its computational efficiency, user-friendly interface, and well-established drift–diffusion framework, which enable rapid parametric optimization and comparative analysis of device architectures. These features make SCAPS-1D particularly attractive for large-scale numerical screening studies, as reflected by its frequent adoption in the analyzed literature. However, the simplified one-dimensional formulation inherently limits its ability to capture complex three-dimensional effects, such as lateral inhomogeneities, spatial dependence, and geometric effects, which play a crucial role in perovskite-based devices under realistic operating conditions. In contrast, multiphysics platforms, such as COMSOL Multiphysics, offer greater flexibility for modeling coupled optical, electrical, and thermal processes, as well as spatially resolved transport dynamics. Despite these advantages, their higher computational cost and increased modeling complexity often restrict their use to a particular case study (ion migration behavior at the interface) rather than large comparative analyses of different materials.

In perovskite solar cells, ion migration is a key factor affecting both performance and long-term stability. Mobile ionic defects, particularly halide ions, can accumulate at grain boundaries and interfaces, altering the local electric field distribution and creating spatially varying internal potentials that influence charge transport and recombination behavior. These effects have been shown experimentally and theoretically to play a dominant role in phenomena such as current–voltage hysteresis and degradation under operation, especially in polycrystalline films where grain boundaries are abundant [51].

One-dimensional drift–diffusion models inherently assume uniform material properties along lateral directions and average out spatial variations across grain boundaries and interfaces. As a result, they cannot explicitly resolve three-dimensional features, such as localized electric field distortions caused by ion accumulation or the impact of inhomogeneous defect distributions and interface roughness on charge transport [52]. These limitations mean that while 1D models are useful for fast comparative studies and screening, they may not fully capture the physical mechanisms of ion migration and interface disorder that critically influence device performance and stability trends.

## 5. Conclusions

The state of the art for computational methods to study solar cells and their electromagnetic behavior under light is presented. Detailed science mapping analysis has been performed for the two different datasets. Using the first dataset, five research questions have been thoroughly answered, especially highlighting the fundamental role of numerical modeling in the field of photovoltaics. Co-authorship analysis has been conducted across countries and organizations to identify emerging trends in this field. Baig Faisal and Ramasamy, P., were found to be the most essential authors with the highest number of documents published. China and the USA are the countries with the highest number of link strengths, representing their strong collaboration in this field. The Chinese Academy of Sciences (CAS) was found to have published a maximum of fifty-eight scientific documents. The twenty most related keywords are discussed, where “efficiency” is found to be the most highly occurring among all the analyzed studies. A focused sub-analysis examined the limitations of current numerical models concerning absorber thickness, ETL/HTL layer properties, and illumination-dependent charge transport. The results highlighted that most existing studies rely heavily on SCAPS-1D simulations, with fewer efforts toward multiphysics modeling that couples optical and electrical domains. In particular, coupling optical finite-difference time-domain (FDTD) simulations with electrical transport models would enable a more accurate description of light absorption profiles, carrier generation, and spatially resolved recombination dynamics. Effective benchmarking for the selection of ETL/HTL materials should prioritize favorable band alignment with the absorber, minimized interfacial recombination losses, adequate charge carrier mobility, and stability under operating conditions, rather than relying solely on absolute efficiency values. In conclusion, future work should aim to integrate three-dimensional coupled opto-electrical models with favorable band alignment to provide a more realistic representation of carrier dynamics at ETL/HTL materials and efficiency optimization in perovskite solar cells.

## Figures and Tables

**Table 1:** List of keywords belonging to each cluster.

Clusters	Number of Keywords	Keywords
1	20	band-gap, buffer layer, cigs, conversion efficiency, czt, dependence, deposition, enhancement, growth, interface, numerical analysis, optical-properties, performance, photovoltaics, scaps-1d, simulation, solar cells, temperature, thickness, thin film
2	18	crystalline silicon, defects, degradation, device modeling, device simulation, efficiency, fabrication, gaas, impact, light trapping, numerical modeling, numerical simulations, parameters, recombination, silicon, silicon solar cells, temperature-dependence model, unified mobility model
3	17	efficient, electron, films, graphene, halide perovskites, hole transport layer, hysteresis, layers, open-circuit voltage, organic solar cells, oxide, perovskite, perovskite solar cells, power conversion efficiency, stability, transport, voltage
4	10	absorption, energy, extraction, identification, model, modeling, modules, optimization, photovoltaic cells, solar energy
5	4	design, heterojunction, high-efficiency, tandem solar cell

**Table 2:** List of the twenty most important keywords based on link strength.

Keyword	Occurrences	Total Link Strength
efficiency	244	513
solar cells	265	442
performance	195	434
simulation	153	358
scaps-1d	110	296
layers	80	220
numerical simulations	130	215
optimization	85	213
thin film	81	204
perovskite solar cells	92	197
recombination	77	195
design	57	138
silicon	69	137
temperature	48	128
photovoltaics	56	119
perovskite	34	117
halide perovskites	33	104
transport	38	103
efficient	32	102
device simulation	42	95

## Data Availability

No new data were created or analyzed in this study. Data sharing is not applicable to this article.
